# Lifetime expectancy and quality-adjusted life-year in Alzheimer’s disease with and without cerebrovascular disease: effects of nursing home replacement and donepezil administration – a retrospective analysis in the Tajiri Project

**DOI:** 10.1186/s12883-015-0475-1

**Published:** 2015-11-05

**Authors:** Kenichi Meguro, Kyoko Akanuma, Mitsue Meguro, Mari Kasai, Hiroshi Ishii, Satoshi Yamaguchi

**Affiliations:** Division of Geriatric Behavioral Neurology, CYRIC, Tohoku University, Sendai, Miyagi 980-8575 Japan; The Osaki-Tajiri SKIP Center, Osaki, Miyagi 989-4413 Japan

**Keywords:** Quality-adjusted life-year, Cerebrovascular disease, Alzheimer’s disease, Donepezil

## Abstract

**Background:**

We previously demonstrated a positive correlation with nursing home (NH) replacement and donepezil (DNP) administration on lifetime expectancy after the onset of Alzheimer’s disease (AD). However, the correlation with quality-adjusted life-year (QALY) remains to be elucidated, along with the additional impact of concomitant cerebrovascular disease (CVD). Based upon our recently reported health state utility values, we retrospectively analyzed the correlation with NH replacement and/or DNP administration on QALY and life expectancy in ‘pure’ AD (without CVD) and AD with CVD patients.

**Methods:**

All outpatients at the Tajiri Clinic from 1999–2012 with available medical records and death certificates were included. The entry criteria were a dementia diagnosis (DSM-IV) and diagnoses of pure AD or AD with CVD (NINCDS-ADRDA), medical treatment for more than 3 months, and follow up to less than 1 year before death. The main outcomes were lifetime expectancy (months between the onset of dementia and death) and QALY.

**Results:**

We identified 390 subjects, of whom 275 had the diagnosis of dementia that met the entry criteria, including 67 pure AD, 33 AD with CVD, and 110 VaD patients. For the AD patients, 52 had taken DNP and 48 had not received the drug due to treatment prior to the introduction of DNP in 1999 in Japan. For the pure AD group, there were positive correlation between NH and DNP and QALY, as well as lifetime expectancy. As for the AD with CVD group, only a correlation between DNP and lifetime expectancy was noted, with no correlation with QALY.

**Conclusions:**

We found positive correlations between DNP administration and NH replacement and lifetime expectancy and QALY after the onset of AD. However, concomitant CVD negated such a positive correlation with QALY. The findings suggest that QALY in AD is affected by CVD; thus, indicating the importance of CVD prevention.

## Background

There are no curative drugs for Alzheimer’s disease (AD) at present; however, symptomatic drugs, such as cholinesterase inhibitors (ChEIs) can delay progression of the disease. This effect, combined with psychosocial interventions, can increase the quality of life (QOL) [1]. These drugs administration can delay nursing home placement [2] and may reduce mortality for patients living in nursing homes [3] and in the community [4]. Beyond delayed progression and improved QOL, the ultimate outcome of drug treatment should be measured in terms of lifetime expectancy.

The correlations of drug therapy with lifetime expectancy have not been fully investigated with various findings. ChEIs were reported to be able to delay a nursing home replacement, but have no effect on lifetime expectancy [4], or ChEIs were associated with a lower risk of death and myocardial infarction [5]. Using the database of all outpatients with available medical records and death certificates at the Tajiri Clinic from 1999–2012, we identified 100 patients that were diagnosed with AD; 52 had taken DNP and 48 patients had not received the drug due to treatment prior to the introduction of donepezil in 1999 in Japan. We previously reported a positive impact of nursing home (NH) placement and DNP administration on lifetime expectancy after the onset of AD [6].

However, simple prolongation of the lifetime expectancy, with possible decreased QOL, remains a problem. Instead, “healthy life expectancy” has emerged as an important issue. In this respect, the quality-adjusted life-year (QALY) and health state utility values (HSUVs) are major QOL scales that are used in the analyses of health economics of diseases [7]. In Japan, the most common dementia disease is AD with cerebrovascular disease (CVD), followed by ‘pure’ AD (without CVD) [8, 9]. With respect to the need to reconsider QALY in the context of activities of daily living (ADL) levels in dementia, we previously calculated the HSUVs under the conditions of AD and AD with CVD: from previous reports and EQ-5D [10–12], we estimated that the HSUVs of pure AD and AD with CVD for ADL level A (independent walking and eating), B (some problems with walking but sitting without assistance), and C (confined to bed) [13] were 0.61 and 0.58, 0.53 and 0.28, and 0.19 and 0.05, respectively [14].

Using the same database, we re-analyzed the correlation between NH replacement and/or DNP administration and QALY and lifetime expectancy in AD without CVD and AD with CVD. We hypothesized that 1) the drug would exhibit a positive correlation with QALY as well as lifetime expectancy in both AD without CVD and AD with CVD patients, and 2) that NH residency would also exhibit a positive correlation. We analyzed DNP alone, because this drug has been used since 1999 in Japan, whereas other drugs, such as galantamine, have only been used since 2011. The combined effect of DNP and NH residency was also analyzed. Despite the retrospective design, this was a long-term study of the possible effect of donepezil on QALY, as well as the life expectancy of patients with AD without CVD and AD with CVD.

## Methods

### Dementia diagnosis

Diagnoses of the following diseases were determined during a meeting of two neurologists, a psychiatrist, and a physician.

1. AD without CVD was diagnosed in patients who met the NINCDS-ADRDA criteria for probable AD [15] and had no CVD on MRI. On MRI, low signal intensity on T1-weighted images, high signal intensity on T2-weighted images, and high signal intensity surrounding the low signal intensity areas on FLAIR images were considered to indicate CVD.
2. AD with CVD was diagnosed according to the NINCDS-ADRDA criteria for probable AD and on evidence for the presence of CVD on MRI; however, CVD lesions were judged to be concomitant with AD and not responsible for cognitive deterioration.

Diagnoses of other dementing diseases were described in the previous report [6].

Written informed consent was obtained from each patient and from the family of those with dementia at entry according to the Declaration of Helsinki (BMJ 1991; 302: 1194). The study was approved by the ethical committee of Tohoku University Graduate School of Medicine, as well as those of the Osaki-Tajiri SKIP Center.

### Analyses

The main outcomes were QALY and lifetime expectancy (i.e., the number of months between the onset of dementia and death). The onset of dementia was confirmed by extensive hearing of medical histories from their families. Two-way ANOVA with the covariance of age and sex, included the effects of NH replacement and DNP administration for the AD and AD with CVD groups. Spearman’s correlations were used to examine the relationship between the DNP use periods and QALY as well as the lifetime expectancy in both groups. Data were available for all 100 participants. The sample size was sufficiently powered to analyze the group effect at a significance level of *p* < 0.05. The error protection was = 0.05 and power was 0.8, thus the effected size was 15 for each group.

## Results

### Demographics

As described previously, of the 100 patients with AD and AD with CVD (both of which being diseases that can be treated with DNP), 52 received DNP and 48 patients did not receive the drug due to treatment prior to the introduction of DNP in 1999 in Japan.

### Effects of NH replacement and DNP administration

Table 1 presents the effects of NH replacement and DNP administration on lifetime expectancy and QALY. For the pure AD group, there were positive SNH and DNP effects on QALY as well as lifetime expectancy. As for the AD with CVD group, there were no NH effects, only the DNP effect on life expectancy with no effect on QALY.

**Table 1:** Effects of NH replacement and DNP administration on lifetime expectancy and QALY

	own homes		NH replacement		F-values			covariance	
	no DNP	DNP	no DNP	DNP	NH	DNP	interact	age	sex
AD	*n* = 25	*n* = 27	*n* = 6	*n* = 7					
lifetime expectancy	56.3 (44.6)	84.7 (36.3)	122.5 (59.7)	156.4 (37.8)	20.442***	4.704*	0.002	0.873	2.482
QALY	26.4 (17.8)	47.0 (20.5)	53.1 (18.2)	75.7 (18.5)	15.123***	11.859***	0.000	0.854	3.738
AD with CVD	*n* = 9	*n* = 14	*n* = 8	*n* = 4					
lifetime expectancy	49.9 (41.6)	77.6 (32.9)	57.5 (44.6)	119.0 (46.2)	2.207	7.861**	1.034	0.265	0.364
QALY	30.4 (21.3)	41.9 (16.1)	30.8 (27.1)	54.4 (29.4)	0.74	3.608	0.65	0.077	2.162

Two-way ANOVAs with the covariance of age and gender were performed

*p < 0.05, **p < 0.01, ***p < 0.001

*AD* Alzheimer's disease, *CVD* cerebrovascular diseases, *QALY* quality-adusted life-year

*DNP* donepezil, *NH* nursing home

On top of the previous analysis (DNP vs Non-DNP) [6], AD with/without CVD were herein analyzed; thus the significant *p*-values would be better less than 0.05/2 = 0.025. The correlation between DNP and lifetime expectancy in the AD without CVD group (F = 4.704) in Table 1 may be influenced by the familywise error.

### Correlations between the periods of DNP use

**Fig. 1:**
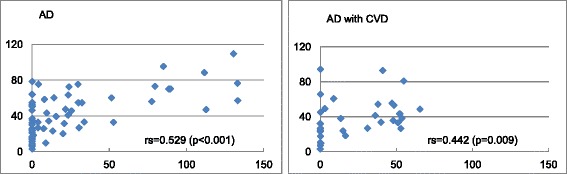
Period of DNP use and QALY for each AD group

Spearman’s correlation analyses revealed that all relationships between the period of DNP use and QALY, as well as lifetime expectancy were significantly positive (biologically meaningful) in the AD and AD with CVD patients. Figure 1 presents the period of DNP use and QALY for each AD group.

## Discussion

### Summary of the results

Our previous study reported the positive effects of DNP administration and NH replacement on lifetime expectancy after the onset of AD; however, simple prolongation of lifetime expectancy with possible decreased QOL remains a problem. Instead, “healthy life expectancy,” i.e., QALY, remains an important issue. Herein, we report the effects on QALY. No covariance effects of age and sex may be due to all patients were old and male to female ratio was not so different between the groups.

### Positive correlation between DNP and/or NH replacement and lifetime expectancy and QALY in pure AD

We previously discussed that while the possible correlation between DNP and mortality remains uncertain, there is also uncertainty regarding its effect on QALY. DNP may have a negative effect on aspiration pneumonia due to the side effect of nausea. An increased gastro-esophageal reflex may also induce pneumonia. This suggests that the effect of DNP on lifetime expectancy or QALY was not purely pharmacological.

DNP administration can improve psychomotor speed or attention function, associated with the frontal lobe [16, 17]. It is consistent with the higher mortality in older adults with lower perceptual speed [18]. Their activities of daily living (ADL) are also stimulated. In a study of the long-term effects of DNP and the use of community-based home help service, the drug was reported to maintain higher self-supported levels of instrumental ADL [19]. Rehabilitation also exhibits a long-term effect in decreasing mortality, and particularly improves motor disability and ADL [20], and prevents aspiration pneumonia [21].

### Concomitant CVD negated the positive correlation with QALY in AD with CVD, but the correlation between DNP and lifetime expectancy remained

However, concomitant CVD negated such positive effects. Each factor is discussed in turn.

The improvement of psychomotor speed or attention function after DNP administration could be poorer in the AD with CVD than in the pure AD patients. By definition, no concomitant CVDs were located in the “strategic” areas, such as the thalamus in the AD with CVD patients; however, the effect of white matter changes on the cholinergic network can be considered. Indeed, the CHIPS scores in these patients were higher than the pure AD patients (data not shown). Thus the effect of DNP on psychomotor speed or attention function is not “full-blown,” and the possible lack of a “synergistic” effect with psychosocial intervention performed in the NH cannot lead to any improvement of QALY.

It is noteworthy that the effect of DNP remained with respect to lifetime expectancy. There were no remarkable differences in the incidence of pneumonia and/or respiratory failure as the cause of death between the AD and AD with CVD groups, and it is only possible to speculate the reason for the effect of DNP on lifetime expectancy in the absence of an effect on QALY. Taking into consideration nausea as a side effect of DNP, together with increased gastro-esophageal reflux, both may induce pneumonia; the effect of DNP on lifetime expectancy was not considered as being purely pharmacological. However, increasing the level of acetylcholine to approach a healthy level in the brain might influence the “energy level” of whole body and thus the biological lifetime would be prolonged. Another possibility is that when the QALY is evaluated based on the HSUV, which is mainly based on ADLs, meaningful but statistically small changes of QALY might be underestimated.

### Socio-economic effects

We calculated QALY of AD and AD with CVD, based on the HSUV for AD by taking ADL into consideration. There were no previous reports that estimated the HSUVs according to the AD severity with ADL extent, or considered complications, including CVD, but only domestic studies on items of AD [22], long-term care [23], and the extent of ADL [24] were found but there were no reports that evaluated each of these issues together. We estimated the HSUV value based on the ADL. We suggest that the analysis of healthcare economics of dementia in Japan could be improved through consideration of the extent of ADL, in addition to the severity of AD.

**Fig. 2:**
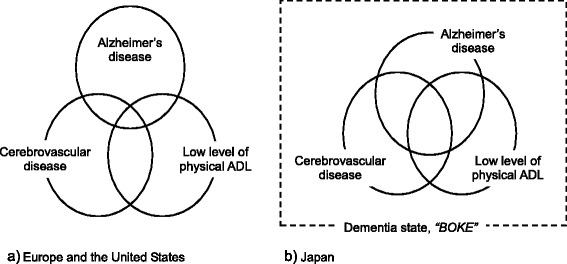
The models of the relationship between Alzheimer’s disease, cerebrovascular disease, and low level of physical ADL in Europe and the United States and Japan

Figure 2 presents our hypothesis of the concept of the dementia state. In Western countries, few mild AD patients also exhibit CVD and low levels of physical ADL. However, in Japan, most mild AD patients could exhibit CVD and low levels of physical ADL. This state is called ‘boke' in Japanese.

The “Orange Plan”, a 5-year plan for the promotion of measures against dementia in Japan, suggests that people with dementia want to live in their own home as long as possible. However, people with dementia or mild cognitive impairment (MCI) face difficulties in maintaining their daily lives. Maintaining a good level of drug compliance remains an essential part in preserving their lives at home. Also, the findings suggest that QALY in dementia is affected by CVD; thus, indicating the importance of CVD prevention. Indeed, the most common dementing disease in Japan is AD with CVD. Comprehensive measures for stroke and dementia prevention (primary, secondary, and tertiary) in the community are necessary to maintain the QOL in older adults [25, 26].

## Conclusions

Although this report has the limitation as all retrospective analyses: i.e., the lack of randomization, we found a positive correlation between DNP administration as well as NH replacement and lifetime expectancy and QALY after the onset of AD. However, concomitant CVD negated such positive correlation with QALY. The findings suggest that QALY in AD is affected by CVD, thus indicating the importance of CVD prevention.
